# The influence of mindfulness-based interventions on the academic performance of students measured by their GPA. A systematic review and meta-analysis

**DOI:** 10.3389/fnbeh.2022.961070

**Published:** 2022-08-26

**Authors:** Thomas Ostermann, Martin Pawelkiwitz, Holger Cramer

**Affiliations:** ^1^Department of Psychology, Faculty of Health, University Witten/Herdecke, Witten, Germany; ^2^Department of Internal and Integrative Medicine, Evang. Kliniken Essen-Mitte, Faculty of Medicine, University of Duisburg-Essen, Essen, Germany; ^3^Institute for General Practice and Interprofessional Care, University Hospital Tuebingen, Tuebingen, Germany; ^4^Bosch Health Campus, Stuttgart, Germany

**Keywords:** academic performance, mindfulness, meta analysis, grade point average, students

## Abstract

**Objective:** Mindfulness-based interventions are increasingly used in health, economic and educational systems. There are numerous studies demonstrating the effectiveness of mindfulness-based interventions in the educational sectors (primary, secondary, and tertiary). This systematic review and meta-analysis assessed the current state of research on the effectiveness of mindfulness-based interventions on the academic performance of students as measured by their grade point average (GPA).

**Methods:** Literature search was conducted in Psychology and Behavioral Sciences Collection, PsycARTICLES, PubMed, and Google Scholar through March 2022. The inclusion criteria were: (1) the use of GPA as a measure of students’ academic performance, (2) a sample that was subjected to a mindfulness-based intervention without medical indication, (3) the student status of the subjects. Meta-analysis was conducted using a random effects model with the generic inverse variance method.

**Results:** The search included a total of 759 studies, of which six randomized controlled trials met the inclusion criteria. In these trials, significant group differences for GPA were found with effect sizes ranging from *d* = 0.16–1.62 yielding a significant overall effect of *d* = 0.42 (95% CI: 0.15–0.69) and a low magnitude of heterogeneity of *I*^2^ = 37%.

**Discussion:** In conclusion, the first results of this emerging research field seem promising. However, the exact mechanisms of action are still unclear.

## Background

One of the strongest predictors of students continuing their studies at university is the Grade Point Average (GPA; DeBerard et al., 2004). As an objective measure of the academic performance of students, by which they are measured even after graduation during their career entry, the average grade is a manageable and, through the documentation of the universities, easily accessible operationalization of academic performance for research.

In many societies, the average grade is used to measure and document the previous school and university performance of pupils and students and is used as a predictor of future performance (Tatar and Düştegör, 2020; Koropanovski et al., 2022) and job satisfaction (Al-Asmar et al., 2021). One reason for this is that one of the best predictors of future performance is past performance. The average grade is also one of the best predictors of the performance of students, which is crystallized in their GPA (Wolfe and Johnson, 1995; McKenzie and Schweitzer, 2001). Besides a person’s intelligence (Duckworth and Seligman, 2005), GPA is also associated with the personality traits like conscientiousness or openness to experience (Mammadov, 2022). Student motivation has a moderating effect on the relationship of openness to experience and conscientiousness with the GPA (Komarraju et al., 2009; Hazrati-Viari et al., 2012). In addition, self-control (Wolfe and Johnson, 1995; Tangney et al., 2018) and self-discipline explain a large proportion of variance in the GPA (Duckworth and Seligman, 2005, 2006). Sleep quality (Trockel et al., 2000; Önder et al., 2014; Nagane et al., 2016), and socioeconomic status are further associated with GPA (White, 1982; Sirin, 2005; Burbidge et al., 2018).

The concept of mindfulness has its historical origin primarily in Buddhism. Mindfulness is often described as a state of consciousness when attention is non-judgmental and is directed to the present moment and the constant development of the phenomena of the present moment. Phenomena can be understood as thoughts, feelings, and body sensations that are allowed to pass without attachment (Kabat-Zinn, 2003). Mindfulness-based interventions thus can be seen as interventions that have internalized this basic idea in their underlying axioms and apply it in its specific form. Yoga, Qi Gong, Tai-Chi, and meditation can be understood as mindfulness-based interventions. The interventions often differ regarding the focus of attention in the exercises. There are systems that focus more on the body (e.g., Yoga and Qi Gong), and there are systems where the mind plays a central role (as in classical sitting meditation).

A currently very popular form of mindfulness-based intervention is the Mindfulness-based stress reduction (MBSR) program by John Kabat-Zinn. The effectiveness of the program, which spans 8 weeks and includes a variety of formal and informal mindfulness-based interventions such as yoga exercises and sitting meditation, has been demonstrated in numerous studies. Its effectiveness covers a wide range of mental and physical illnesses. There are a large number of studies that prove its effectiveness in chronic pain, cancer, heart disease, depression, and anxiety disorder (Grossman et al., 2004). Dispositional mindfulness has e.g., been shown to be negatively associated with suicidal ideation (Lamis and Dvorak, 2014). Moreover, mindfulness moderates the association between affective temperaments and psychiatric symptoms (Barnhofer et al., 2011). This is important, because, affective temperaments are independently and more strongly associated with negative clinical outcomes than a diagnosis of a major affective disorder (Baldessarini et al., 2017).

Mindfulness has also found its way into the educational system, its application can be found in primary, secondary, and tertiary education (Reber, 2014). Outcomes include social, health, and work/performance (Shapiro et al., 1998; Beauchemin et al., 2008). Although further studies are needed in this field, it seems that the effects of mindfulness-based interventions in pupils and students differ during their development span (Reber, 2014).

This systematic review and meta-analysis aimed to assess the current state of research on the effectiveness of mindfulness-based interventions on the academic performance of students as measured by their grade point average (GPA). Thus, the main hypothesis was that students’ GPA will be higher after mindfulness interventions compared to a non-mindfulness control group.

## Methods

The review was planned and conducted in accordance with PRISMA guidelines for systematic reviews and meta-analyses (Moher et al., 2009) and the recommendations of Cochrane (Higgins et al., 2019).

### Eligibility criteria

The inclusion criteria of this systematic review and meta-analysis were: (1) the use of GPA as a measure of students’ academic performance, (2) a randomized study design with at least one group that was subjected to a mindfulness-based intervention without medical indication, (3) the student status of the subjects. Exclusion criteria were whether: (1) the GPA was measured as an indicator for the academic performance of the students, (2) a sample of students without medical indications such as depression, ADHD, PTSD, clinically relevant anxieties, etc. was selected, and whether (3) the study was conducted with students since the English word “students” denotes both students and pupils. Exact duplicates were excluded from the results. The decision to exclude studies was made by consensus with an external expert. No restrictions were used for the primary data search, e.g., the publication date. Due to the linguistic knowledge of the persons involved in the review, only German and English studies could be considered.

### Literature search

The following electronic databases were used for the primary study search on March 08, 2022: Psychology and Behavioral Sciences Collection, PsycARTICLES, PubMed. The following search terms were used: students AND mindfulness AND (GPA or grade point average or academic achievement or academic performance).

The search terms were defined *a priori* by consensus with an external expert. In addition, a search with the search terms defined at the beginning was carried out in the Google Scholar search engine and added as an additional source. For Google Scholar the search terms “college students” mindfulness “grade point average” “student success” “control group” were used.

Web of science, Scopus, and ScienceDirect were not searched separately because Google Scholar has been shown to find nearly all citations covered by these databases (Martín-Martín et al., 2018).

The studies found in this way were checked according to the eligibility criteria for fit with the orientation of the review. The title, abstract, and full text of the retrieved studies were examined for their fit with the orientation of the review.

### Risk of bias in individual studies

Two reviewers independently assessed the risk of bias in individual studies using the Cochrane risk of bias tool. This tool assesses the risk of bias on the following domains: selection bias, performance bias, attrition bias, reporting bias, and detection bias (Higgins et al., 2019). Again, discrepancies were rechecked with a third reviewer, and a consensus was achieved by discussion. If necessary, trial authors were contacted for further details.

### Data analysis

Meta-analysis was conducted using Review Manager Software (Version 5.3, The Nordic Cochrane Centre, Copenhagen) by random effects model using the generic inverse variance method. Cohen’s d with the standard error was calculated as the difference in post-intervention means or pre to post change scores between groups divided by the pooled standard deviation. Where no standard deviations were available, Cohen’s d was calculated from t-, F- or z-statistics (Lakens, 2013). The magnitude of heterogeneity was analyzed using the *I*^2^ statistics and categorized as (1) *I*^2^ = 0%–24%: low; *I*^2^ = 25%–49%: moderate; *I*^2^ = 50%–74%: substantial; and *I*^2^ = 75%–100%: considerable heterogeneity.

## Results

### Literature search

**Figure 1:**
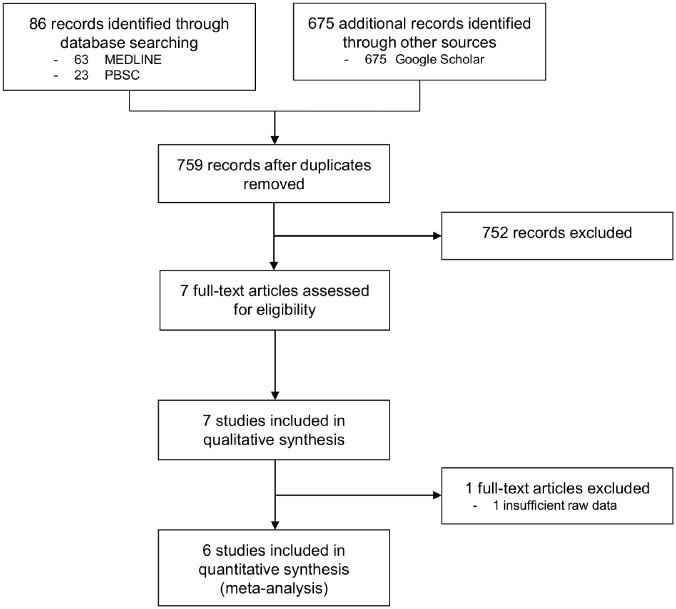
Flowchart of the results of the literature search.

In the primary data search in the databases, PBSC and MEDLINE 86 records were found. The search in the Google Scholar database revealed 675 additional records. After examination of the exclusion criteria, seven studies were included in the qualitative, and six studies in the quantitative analysis (Figure 1).

### Study characteristics and findings

In the following, the studies are presented in a modified form of the PICO scheme in a tabular overview (Table 1). The risk of bias in individual studies is reported in Table 2.

**Table 1:** Tabular overview of the characteristics of the included RCTs (EG: experimental group; CG: control group).

Author (Year)	Origin	Participants	n (EG/CG)	Intervention	Control	Main results on GPA
Baumgartner and Schneider (2021)	USA	College students	27/29	7-week MBSR intervention without full day silent mindfulness practice at the end	1. No treatment 2. Study skills group	Significant difference (*d* = 1.62 compared to no treatment; *d* = 1.41 compared to study skills group).
Butzer et al. (2015)	USA	9th or 10th grade physical education class at a public high school	44;51	12-week school-based yoga intervention (28/29 sessions of 35–40 min)	Physical education as usual (28 sessions of 35–40 min)	Significant difference (*d* = 0.13).
Güldal and Satan, (2020)	Turkey	Students of the 10th grade from a religious high school for girls	10;10	8-week mindfulness based psychoeducation program	Regular guidance lessons including learning styles, study habits, and self-esteem	No significant difference (*d* = 0.16).
Eswari (2018)	Bahrain	Students with an undergraduate degree in science subjects	59;59	Yoga and meditation training including physical postures, breathing techniques	No treatment	Significant difference (*d* = 0.26).
Hall (1999)	USA	Undergraduate students from an introductory psychology course	28;28	Meditation training including natural breathing techniques, relaxation, and attention-focusing techniques practiced for a duration of 10 min at the start and end of each study session	No treatment	Significant difference during (*d* = 0.55) and after the semester (*d* = 0.68).
Sampl et al. (2017)	Austria	Bachelor students	51; 58	Two hour group MBSLT intervention for 10–15 participants over a time period of 10 weeks	No treatment	Significant difference (*d* = 0.64).

**Table 2:** Risk of bias assessment of the included studies using the Cochrane risk of bias tool.

Bias						
Reference	Random sequence generation (selection bias)	Allocation concealment (selection bias)	Blinding of participants and personnel (performance bias)	Blinding of outcome assessment (detection bias)	Incomplete outcome data (attrition bias)	Selective reporting (reporting bias)	Other bias
Baumgartner and Schneider (2021)	Low risk	Unclear risk	Unclear risk	Unclear risk	High risk	Low risk	Low risk
Butzer et al. (2015)	Unclear risk	Unclear risk	Unclear risk	Low risk	Unclear risk	Low risk	Unclear risk
Güldal and Satan (2020)	Unclear risk	Unclear risk	Unclear risk	Unclear risk	Low risk	Low risk	Unclear risk
Eswari (2018)	Unclear risk	Unclear risk	Unclear risk	Unclear risk	Low risk	Low risk	Unclear risk
Hall (1999)	Unclear risk	Unclear risk	Unclear risk	Unclear risk	Unclear risk	Low risk	Unclear risk
Sampl et al. (2017)	Unclear risk	Unclear risk	Unclear risk	Unclear risk	Unclear risk	Low risk	Low risk

In addition, the studies and their results are presented alphabetically according to the name of the first author.

A recent randomized controlled trial of Baumgartner and Schneider (2021) investigated the impact of a 7-week MBSR intervention without a full day of silent mindfulness practice at the end on academic resilience and performance in 47 college students compared to an active control group of 52 students and an untreated control group of 29 students. Participants completed daily meditation or study logs. GPA was obtained for the semester prior to the study and of the study semester. Using a repeated-measures ANCOVA adjusting for pre-intervention found significant GPA improvements in the MBSR-group, while GPA remained unchanged in the control groups (*d* = 1.41 compared to active control; *d* = 1.62 compared to untreated control).

Butzer et al. (2015) conducted a study in 9th and 10th grade students on the effects of a 12-week school-based yoga intervention on changes in GPA. Participants included high school students who had registered for physical education randomized to receive either a yoga intervention (*n* = 44) or physical education (*n* = 51). The yoga intervention was designed as a 12-week school-based yoga intervention (28/29 sessions of 35–40 min) while physical education as usual consisted of 28 sessions of 35–40 min. GPA was collected *via* school records at the end of the school year. Results revealed that GPA differed between the yoga and control groups over time (*d* = 0.13) without being significant.

In a randomized controlled trial, 108 students from four study programs were randomized into an experimental group and a control group (Eswari, 2018). The experimental group met three times a week for 90 min to prepare for their studies and exam performances after a 45-min mindfulness training with physical postures, breathing techniques, and meditative exercises. The control group met for the same duration and frequency only to work towards their exams and to discuss current topics and doubts in the study subject. The subjects were obliged to maintain secrecy regarding the instructions. Significant differences in the average GPA favoring the experimental group (GPA = 8.72) over the control group (GPA = 8.51) were found after the intervention (*p* = 0.031; *d* = 0.26).

In a mixed-method research project, Güldal and Satan (2020) conducted a nested in a randomized clinical trial of a 8-week mindfulness-based psychoeducation program compared with regular guidance lessons including learning styles, study habits, and self-esteem in 20 female students of a religious high school for girls in Istanbul equally split to both groups. A difference between the test and control groups’ GPA was found without being statistically significant (*z* = −0.378 *d* = 0.16).

Pamela D. Hall (1999) examined 56 African American students who had previously been randomly assigned to an introductory course in psychology at Hampton University. While the experimental group met once a week for one semester to learn 1 h after they jointly conducted a 10-min mindfulness-based intervention, the control group met to learn without the prior intervention. All subjects were obliged to not divulge to the members of the other group about what was going on in their group. The intervention was a meditation containing elements of natural breathing technique, relaxation, and attention-focusing. The subjects of the experimental group were instructed to use the same process when learning independently for an exam and before taking a test. Statistically significant differences were found between the groups in the GPA during (experimental group GPA = 2.85; control group GPA = 2.55; *p* = 0.041; *d* = 0.55) as well as after the semester were the intervention took place (experimental group GPA = 2.93; control group GPA = 2.48; (*p* = 0.014; *d* = 0.68).

A total of 109 students from the University of Innsbruck were examined in a longitudinal randomized control study (Sampl et al., 2017). The experimental group consisted of 51 subjects (38 women, mean age = 21.39 years), and the control group of 58 subjects (44 women, mean age = 23.07). None of the volunteers or their first-degree relatives had a psychiatric diagnosis. The intervention mindfulness-based self-leadership training (MBSLT) was developed specifically for this study and conducted in a group setting of 10–15 subjects over a period of 10 weeks for 2 h per week. The intervention itself consists of combined elements of the MBSR method (Kabat-Zinn, 1994, 2013) and training proposals for developing the ability of self-leadership (Neck and Manz, 2013). The weighted GPA of the experimental group (GPA = 2.20) was significantly higher in the intervention than in the control group (GPA = 1.78) at the end of the semester (*d* = 0.64).

**Figure 2:**
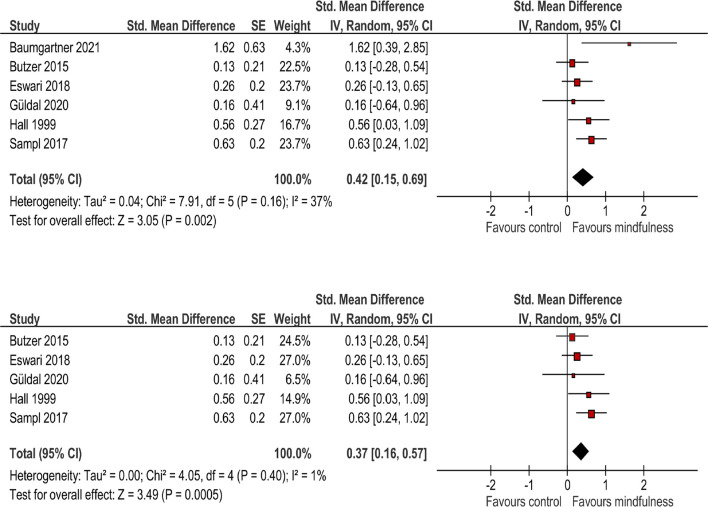
Analysis of overall effect (above) using the complete sample (above) and excluding an outlier (below).

Meta-analysis of these studies revealed a significant overall effect of SMD = 0.42 (95%CI = 0.16–0.88) with moderate heterogeneity. Due to the high effect size of the study of Baumgartner and Schneider (2021), this study was excluded resulting in a reduced heterogeneity without substantively changing the overall effect (Figure 2).

## Discussion

### Summary of findings

Of the 759 studies examined, six RCTs were identified as relevant for answering the question “Do mindfulness-based interventions influence the academic performance of students measured by their grade point average?”. Of these, three studies revealed a statistically significant effect between the experimental group and the control group on the GPA of the subjects (Hall, 1999; Sampl et al., 2017; Baumgartner and Schneider, 2021). No significant difference between the groups was found in the remaining three studies, all of which partly found a meaningful correlation to elucidate the variance of the GPA within the group by at least one measure associated with mindfulness. Another observational study not included in the meta-analysis found a positive correlation within the group studied (Wallace et al., 1984).

### Discussion of findings

The research field of mindfulness-based interventions and their influence on the academic performance of students is of growing importance. Nevertheless, there are some gaps in the study landscape. Looking at the study landscape as a whole, there are a number of contexts that should be taken into account in the evaluation: with regard to the population, a sample selection bias can be assumed. Even though the studies used a randomized design, it is not clear whether these samples are representative of the population of all students to be investigated. Research in this area faces the difficulty of examining a sample representative of the population (all students). It is questionable whether students who willingly implement a regular mindfulness practice in their lives over a longer period and use the necessary resources to do so are representative of the group of all students. Due to the spiritual background of mindfulness-based interventions, it is also unclear whether they can easily be applied to individuals without a spiritual background or from other spiritual traditions. Some studies have already pointed to the influence of these variables (Grossman and Van Dam, 2001; Napora, 2013).

The different expressions of personality traits may be another reason why a group of students that is not representative of the population as a whole tends to be more concerned with mindfulness-based interventions. Mindfulness, for example, seems to be associated primarily with the manifestations of neuroticism and conscientiousness in the Big Five model (Goldberg, 1990). Whereas the relationship between conscientiousness and mindfulness seems to be unclear—and almost unnoticed (Giluk, 2009)—so far, the literature discusses moderating and mediating influences of mindfulness on behavior and experience associated with neuroticism, such as subjective well-being (Wenzel et al., 2015) or the associated development of depressive symptoms and trait anger (Feltman et al., 2009).

Against the background that trait mindfulness has a conspicuous relationship with the personality traits neuroticism and conscientiousness—but also with a high degree of openness to experience (van den Hurk et al., 2011) , and assuming that the personality traits in the sense of the Big Five are almost normally distributed in the entire population, it is questionable that the group of students who function as a sample in the studies that either already has previous experience with (regular) mindfulness practice or are motivated to do so is representative of the total population of all students.

In reference to already expressed assumptions, such as those of Carsello and Creaser (1978), which refer to their own studies and those of Lazarus (1976), the motivation of the students—and the possibly concrete aim to improve their own grades with the intervention—as well as the personality constitution seem to have an influence on the effectiveness of mindfulness-based interventions. These interrelationships have been given little consideration in the studies to date.

Regarding the study structure, it is noticeable that the importance of the person guiding meditation in the research setting as provided in a study of Bambacus (2018) was only marginally discussed in the RCTs included in our meta-analysis. It can be assumed that the instructor in his leading function influences the results of the intervention through his previous experiences and expectations. Similarly, the use of a detailed psychometric test battery, as given in Napora 2017 was only provided in one study (Sampl et al., 2017).

Looking at the interventions, their heterogeneity is noticeable: one study used Transcendental Meditation (TM; Hall, 1999), and three used different programs with elements of MBSR (Sampl et al., 2017; Güldal and Satan, 2020; Baumgartner and Schneider, 2021), two used yoga and meditation (Butzer et al., 2015; Eswari, 2018). It is unclear to what extent the results of different forms of mindfulness-based interventions can be compared. The same is true for the density and duration of the interventions, which also clearly differed between the included studies. There does not seem to be sufficient research on what dose, i.e., what duration, frequency, and length, is necessary to achieve positive effects through mindfulness-based interventions (Lam, 2016; Bambacus, 2018). This is of particular importance given that dose is one of the most important decisions in the implementation of behavioral interventions (Voils et al., 2012).

Taking all this into consideration, the calculated effect size of the randomized controlled trials (*d* = 0.42), which can be understood as evidence of a small effect (Cohen, 1988), can be interpreted as the first hint of cause-effect relationships that should be researched by further studies in the future.

### Limitations

First, this review is subject to the same limitations as other reviews in this field: publication bias or file drawer problem (Rosenthal, 1979; Creswell and Lindsay, 2014). Second, only one person carried out a literature search and study inclusion. Due to the small number of studies at the beginning of the literature search, it was found to be sufficient to have the process carried out carefully by one person, to discuss the process and the results with an external expert, and to evaluate them as adequate and valid for the study situation. After internal discussion, it was decided not to use an evaluation scale but to describe the studies in more detail. This nevertheless represents a limitation of the review, since such scales provide evaluation criteria of comparatively high-quality, which increase the quality of the evaluation of the studies in the review and thus increase the quality of the review.

### Further directions

In future studies, researchers in this field are recommended to take a more detailed account of the demographic data of the subjects, to publish the descriptive statistics relating to the sample and thus check for possible confirmatory variables. This is important because it is essential that the gender distribution, age, and previous study length be surveyed and taken into account. Above all, it must be taken into account that students in their first year are particularly under the influence of stressors and that there will probably be a natural development of academic performance in this period, which should be differentiated from the effect of the intervention. Similarly, ethnicity should be considered (Toomey and Anhalt, 2016). In the future, it will be necessary to check whether the selected sample is representative of the population to be examined. In this sense, further studies should be carried out on the interrelationship between personality traits and mindfulness-based practices. On the other hand, the students’ motivation and expectations in carrying out mindfulness-based practices and whether and in what form previous experiences exist should be taken into account. Accordingly, the health status, the embedding in a religious value system, and previous resources and obstacles in relation to one’s own academic performance should be surveyed. With regard to the health status, it can be assumed that an improvement in symptomatology, which impairs performance, leads to better performance.

The investigation of the mechanisms of action should also be accelerated. Special attention should be paid to the question of type, dose, and timing of the intervention. A detailed test battery should be used that controls underlying variables (such as test anxiety), collects trait mindfulness, and correlates its developments with those of academic achievement. The research process showed that there are some studies that have collected the GPA through the students’ independent data. It is recommended to use university documents instead in order to keep the probability of incorrect information small. It is also recommended to use the cumulative GPA of the second semester of the intervention. In order to adequately correlate the performance of the subjects in the experimental and control groups, it is important to weigh the grades according to the number of examinations taken and the number of credit points collected during the study period. It is recommended to take these indicators into account in the future and to publish them as well.

## Conclusions

The present meta-analysis found a significant group difference in the GPA with a small but significant effect size due to mindfulness-based intervention. Thus, a preliminary indication of the effectiveness of mindfulness-based interventions on the academic performance of students measured against their GPA can be assumed. Therefore, implementing mindfulness training in students’ curricula seems promising. Universities should explore ways to foster a mindful environment to maximize academic prospects for their students. However, further research is needed to support these emerging data and to clarify how this influence is structured.

## Data Availability Statement

The original contributions presented in the study are included in the article, further inquiries can be directed to the corresponding author.

## Author Contributions

TO and MP initiated the project and wrote the first draft. MP and HC conducted the literature search, reviewed and examined the articles. HC and TO extracted the data of the included articles and conducted the meta analysis. MP, HC, and TO wrote, edited, reviewed, and approved the final manuscript. All authors contributed to the article and approved the submitted version.

## Conflict of Interest

The authors declare that the research was conducted in the absence of any commercial or financial relationships that could be construed as a potential conflict of interest.

## Publisher’s Note

All claims expressed in this article are solely those of the authors and do not necessarily represent those of their affiliated organizations, or those of the publisher, the editors and the reviewers. Any product that may be evaluated in this article, or claim that may be made by its manufacturer, is not guaranteed or endorsed by the publisher.
